# Induction of Apoptosis and Cytotoxicity by Isothiocyanate Sulforaphene in Human Hepatocarcinoma HepG2 Cells

**DOI:** 10.3390/nu10060718

**Published:** 2018-06-04

**Authors:** Saie Brindha Kntayya, Muhammad Din Ibrahim, Nooraini Mohd Ain, Renato Iori, Costas Ioannides, Ahmad Faizal Abdull Razis

**Affiliations:** 1 UPM-MAKNA Cancer Research Laboratory, Institute of Bioscience, Universiti Putra Malaysia, 43400 UPM Serdang, Selangor, Malaysia; saiebrindhak@yahoo.co.uk (S.B.K.); marc_dean89@yahoo.com (M.D.I.); noorainim@upm.edu.my (N.M.A.); 2 Consiglio per la Ricerca in Agricoltura e L’analisi Dell’economia Agraria, Centro di Ricerca Agricoltura e Ambiente (CREA-AA), Via di Corticella 133, 40128 Bologna, Italy; renato.iori48@gmail.com; 3 Faculty of Health and Medical Sciences, University of Surrey, Guildford, Surrey GU2 7XH, UK; c.ioannides@surrey.ac.uk; 4 Laboratory of Molecular Biomedicine, Institute of Bioscience, Universiti Putra Malaysia, 43400 UPM Serdang, Selangor, Malaysia; 5 Laboratory of Food Safety and Food Integrity, Institute of Tropical Agriculture and Food Security, Universiti Putra Malaysia, 43400 UPM Serdang, Selangor, Malaysia; 6 Department of Food Science, Faculty of Food Science and Technology, Universiti Putra Malaysia, 43400 UPM Serdang, Selangor, Malaysia

**Keywords:** glucoraphenin, sulforaphene, HepG2 cells, apoptosis, cell cycle arrest

## Abstract

Glucoraphenin, a glucosinolate present in large quantities in radish is hydrolysed by myrosinase to form the isothiocyanate sulforaphene, which is believed to be responsible for its chemopreventive activity; however, the underlying mechanisms of action have not been investigated, particularly in human cell lines. The aim of the study is to assess the cytotoxicity of sulforaphene in HepG2 cells and evaluate its potential to enhance apoptosis. The cytotoxicity of sulforaphene in HepG2 cells was carried out ensuing an initial screening with two other cell lines, MFC-7 and HT-29, where sulforaphene displayed highest toxicity in HepG2 cells following incubation at 24, 48 and 72 h. In contrast, the intact glucosinolate showed no cytotoxicity. Morphological studies indicated that sulforaphene stimulated apoptosis as exemplified by cell shrinkage, blebbing, chromatin condensation, and nuclear fragmentation. The Annexin V assay revealed significant increases in apoptosis and the same treatment increased the activity of caspases -3/7 and -9, whereas a decline in caspase-8 was observed. Impairment of cell proliferation was indicated by cell cycle arrest at the Sub G_0_/G_1_ phase as compared to the other phases. It may be concluded that sulforaphene, but not its parent glucosinolate, glucoraphenin, causes cytotoxicity and stimulates apoptosis in HepG2 cells.

## 1. Introduction

Cancer is one of the leading causes of mortality throughout the world today. Being an extremely complex disease, treating cancer has become a major challenge. There are more than 200 types of cancers, one of which is hepatocellular carcinoma, reported to be amongst the most typically diagnosed cancers [1,2,3].

**Figure 1:**
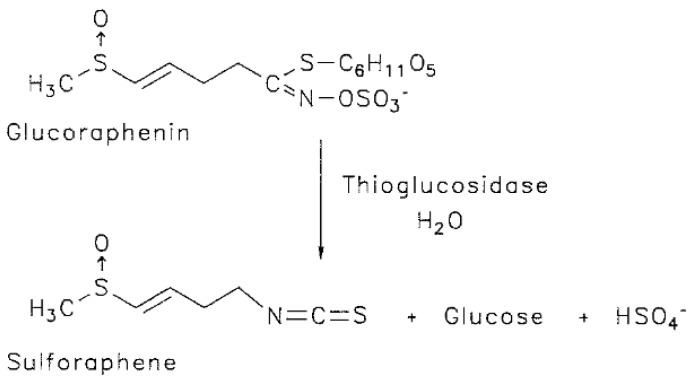
Enzymatic hydrolysis of glucoraphenin to sulforaphene. Adapted from Brinker and Spencer [17].

A number of phytochemicals have been considered as potential cancer chemopreventive agents, e.g., glucosinolates (GLs) (Figure 1), major components found in cruciferous vegetables which over the years have been identified as potential anti-carcinogens against many common cancers [4,5,6,7,8]. GLs are readily hydrolysed upon tissue damage by the myrosinase enzyme into several active compounds, including isothiocyanates that are believed to be responsible for the chemopreventive activity reported in many studies [9,10,11,12,13]. These compounds bring about beneficial changes in cancerous cells providing convincing evidence that isothiocyanates can be potentially developed into anticancer agents of therapeutic benefit to humans. However, the mechanisms of action of these compounds towards the inhibition of cancerous cell growth are still not entirely understood.

Glucoraphenin (GRE, 4-Methylsulfinyl-3-butenyl GL), one among over 200 existing GLs [14], is an aliphatic sulphur containing GL which coexists along with several other GLs in the sprouts of radish, *Raphanus sativus* L. It was reported that GRE and another GL, glucoraphasatin, are among the most promising GLs due to them both bearing an extra sulphur function in their aglycon [7]. The isothiocyanate (Figure 1) derived from the enzymatic hydrolysis of GRE, sulforaphene (4-Methylsulfinyl-3-butenyl isothiocyanate) has in recent years, captured the imagination of researchers because of its potential to function as an anti-cancer agent and to afford protection against several other chronic diseases [5,15]. According to Ippoushi et al. [16], sulforaphene possesses antioxidant properties that are likely to contribute to its cancer chemopreventive activity.

Beevi et al. [6] reported growth inhibition and induction of cell death in two human cancer cell lines following incubation with extracts of *Raphanus sativus* L. The objectives of the present study are to evaluate whether GRE or sulforaphene are responsible for the observed inhibition in cell growth and to investigate whether an increase in apoptotic activity is involved. Such knowledge may lead to genetically modified radishes capable of producing higher levels of these compounds.

## 2. Materials and Methods

### 2.1. Isolation of Glucoraphenin (GRE)

GRE was purified at CREA-AA (ex CRA-CIN), Bologna, Italy, through a collaborative study. The isolation and characterisation of the GL were performed according to Barillari et al. [7]. Briefly, 35 g (dry weight) of *R. sativus* defatted seeds were extracted with 500 mL boiling ethanol 70% (*v*/*v*), homogenised by a U-Turrax (IKA T25) homogeniser (15 min at 75 °C) and centrifuged (15,300× *g* at 4 °C for 30 min). Solid residue was re-extracted with 500 mL of 70% boiling ethanol and centrifuged once again. Extracts were filtered and were then loaded onto an open preparatory column (25 × 200 mm i.d., Pharmacia) containing DEAE-Sephadex A-25 conditioned with 25 mM acetate buffer at pH 5.6. The column was washed with starting buffer followed by formic acid/2-propanol/water (3:2:5) solution and finally buffer again. The column was eluted stepwise with 5 × 100 mL aqueous K_2_SO_4_ (25 mM) and then with 2 × 135 mL K_2_SO_4_ (50 mM). Each fraction was tested for GL content by HPLC. Fractions containing >95% GRE were pooled and concentrated to one tenth of the initial volume. Inorganic salts were precipitated out using absolute ethanol before being freeze-dried. The purity was further improved by gel-filtration removal of contaminants, which was performed using an XK 26/100 column packed with Sephadex G10 chromatography media (Amersham BioSciences, Buckinghamshire, UK), connected to an FPLC System (Pharmacia, Kent, UK). The GL containing sample was dissolved in water (400 mg/mL), and 2 mL was loaded onto a column. The mobile phase was water at a flow rate of 2.0 mL min^−1^, and the eluate absorbance was monitored at 254 nm. Individual fractions were analysed by HPLC. Fractions containing pure GRE were pooled and freeze-dried until further use.

### 2.2. Cell Culture

MCF-7 (HTB-22, oestrogen receptor-positive human breast adenocarcinoma cells), HepG2 (HB-8065, human hepatocellular carcinoma cells) and HT-29 (HTB-38, human colon adenocarcinoma cells) were obtained from American Type Culture Collection (ATCC, Manassas, VA, USA). The MCF-7 and HepG2 cells were maintained in RPMI-1640 medium (Sigma-Aldrich, Munich, Germany), supplemented with 10% sterile-filtered fetal bovine serum (FBS) (Sigma-Aldrich, Germany) and 1% antibiotic (l-glutamine-penicillin-streptomycin) (Sigma-Aldrich, Germany) solution. HT-29 cells were maintained in DMEM medium (Sigma-Aldrich, Germany), supplemented with 10% sterile-filtered fetal bovine serum (FBS) (Sigma-Aldrich, Germany) and 1% antibiotic (l-glutamine-penicillin-streptomycin) (Sigma-Aldrich, Germany) solution. Cells were grown at 37 °C in a humidified incubator containing 5% CO_2_.

### 2.3. Cytotoxicity Assay of GRE, Sulforaphene and Cisplatin

The 3-[4,5-dimethylthiazol-2-yl]-2,5-diphenyltetrazolium bromide (MTT) assay was performed according to the method by Mosmann [18]. For the initial screening, confluent HepG2, MCF-7 and HT29 cells were seeded into three 96-well plates at 1 × 10^5^ cells/mL and incubated for 24 h in a humidified atmosphere of 5% CO_2_ at 37 °C. Cells were treated with serum free medium containing varying concentrations of GRE (0–100 μM) by serial dilution. An amount of 5 μL of myrosinase enzyme (Sigma Aldrich, St. Louis, MO, USA) (0.3 units/mL) was added to each well for conversion of GRE to sulforaphene. Cells were then incubated for 24 h in a humidified atmosphere of 5% CO_2_ at 37 °C. After treatment, the cells were incubated with 20 μL of MTT (Sigma Aldrich, Germany) solution (5 mg/mL in PBS) for 2–4 h in the dark in a humidified atmosphere of 5% CO_2_ at 37 °C. Finally, 100 μL of DMSO was added to each well. The absorbance was measured at 570 nm using an ELISA reader (Tecan, Austria). After screening, for further analysis, only HepG2 cells were selected due to their highest sensitivity towards the compound compared to the two other cell lines. The MTT assay was then carried out on HepG2 cells in a time dependent manner for 24, 48 and 72 h with GRE, sulforaphene and cisplatin (positive control). Results are expressed as percentage cell viability at 24, 48 and 72 h. The growth inhibition of the test agent is expressed as the IC_50_ value.

### 2.4. Morphological Assessment of Apoptotic Cells by TUNEL Assay

The terminal deoxynucleotidyl transferase dUTP nick end labelling (TUNEL) assay was carried out using the DeadEnd^TM^ colorimetric apoptosis detection system (Promega, Madison, WI, USA) according to the manufacturer’s protocol. HepG2 cells were seeded into 25 mL culture flasks at approximately 1 × 10^6^ cells/mL and incubated for 24 h in a humidified atmosphere of 5% CO_2_ at 37 °C. Cells were treated with sulforaphene (in serum free media) at IC_50_ concentration of 72 h (33.8 μM). The cells were incubated for 24, 48 and 72 h in a humidified atmosphere of 5% CO_2_ at 37 °C. Treatment-free cells were grown as a negative control. After incubation, cells were harvested, added onto poly-l-lysine coated slides and air dried. The cells were then fixed with 4% paraformaldehyde solution in PBS for 25 min at room temperature. After fixing, the slides were washed with fresh PBS for 5 min and the cells were permeabilised by immersing the slides in 0.2% Triton^®^ X-100 solution in PBS for 5 min. The cells were washed again with fresh PBS, mixed with 100 μL equilibration buffer and allowed to equilibrate at room temperature for 10 min. rTdT reaction mix (100 μL) was added to the equilibrated areas, covered with plastic cover slips and left to incubate at 37 °C for 60 min. To terminate the reactions, the slides were immersed in 2X SSC for 15 min at room temperature then washed with fresh PBS for 5 min. Slides were immersed in 0.3% hydrogen peroxide in PBS for 5 min to block endogenous peroxidases. The slides were again washed with PBS and mixed with 100 μL Streptavidin HRP solution per slide, and incubated for 30 min. Finally, 100 μL of DAB solution was added to each slide and allowed to stand until a light brown background appeared. Slides were subsequently viewed under a light microscope (Olympus, Tokyo, Japan) to detect morphological changes.

### 2.5. Morphological Assessment of Apoptotic Cells by DAPI Staining

4′,6-diamidino-2-phenylindole (DAPI) staining was carried out according to the method described by Papi et al. [19] with slight modifications. HepG2 cells were grown on sterile glass slides overnight and treated for 24 h with sulforaphene (in serum free media) at IC_50_ concentration of 72 h (33.8 μM). The cells were incubated for 24, 48 and 72 h in a humidified atmosphere of 5% CO_2_ at 37 °C. At the end of the incubation, cells were fixed with 4% paraformaldehyde and then permeabilised with Triton X-100 (0.1% in PBS). Cells were finally stained using DAPI in PBS (2.5 μg/mL) and allowed to stand for 20 min in a dark condition. Finally, morphological changes were viewed using fluorescence microscopy (Zeiss, Oberkochen, Germany).

### 2.6. Morphological Assessment of Apoptotic Cells by Acridine Orange (AO) Propidium Iodide (PI) Double Staining

The AO/PI morphological assessment of apoptotic cells was carried out according to the method of Arbab et al. [20] with minor modifications. This double-staining method was performed on HepG2 cells treated with sulforaphene for 24, 48 and 72 h, and examined by fluorescence microscopy. HepG2 cells were seeded into 25 mL culture flasks at approximately 1 × 10^6^ cells/ mLand incubated for 24 h in a humidified atmosphere of 5% CO_2_ at 37 °C. The cells were treated with sulforaphene (in serum free media) at IC_50_ concentration of 72 h (33.8 μM). The cells were incubated for 24, 48 and 72 h in a humidified atmosphere of 5% CO_2_ at 37 °C. Treatment-free cells were grown as a negative control. Cells were harvested with trypsin (Sigma-Aldrich, Germany), centrifuged at 300× *g* for 10 min and kept cool on ice. Separately, 5 μL AO (10 mg/mL) was mixed with 5 μL PI (10 mg/mL) making a double-staining dye of AO/PI and kept in an ice bath in dark conditions. An aliquot of the cell suspensions (10 μL) was added to the dye mixture and transferred (5 μL) onto a glass slide for fluorescence viewing. Slides were observed under a UV-Fluorescence microscope within 30 min.

### 2.7. Annexin V-Fitc Assay

The Annexin V-FITC assay was carried out using the Annexin V-FITC assay kit (Sigma-Aldrich) according to the protocols provided. Briefly, HepG2 cells were seeded into 25 mL culture flasks at approximately 1 × 10^6^ cells/ mL and incubated for 24 h. Cells were treated with sulforaphene (in serum free media) at an IC_50_ concentration of 72 h (33.8 μM). The cells were treated for 24, 48 and 72 h in a humidified atmosphere of 5% CO_2_ at 37 °C. Treatment-free cells were grown as negative controls. The cells were harvested with trypsin and centrifuged at 300× *g* for 10 min after treatment. The cells were then washed in PBS three times to remove any remaining media. Annexin V-FITC (5 μL) was added to 195 μL of cell suspension binding buffer (50 mL binding buffer and 150 mL distilled water), mixed and incubated in the dark for approximately 10 min. The cells were then washed and resuspended in 190 μL binding buffer (50 mL binding buffer and 150 mL distilled water) and finally 10 μL PI solution was added. The cells were kept on ice and then analysed by flow cytometry (Becton Dickinson, Franklin Lakes, NJ, USA)

### 2.8. Caspase-3/7, -8 and -9 Activity Assay

The activities of caspase-3/7, -8 and -9 were measured using a luminescence assay, with Caspase-Glo^TM^ 3/7, Caspase-Glo^TM^ 8 and Caspase-Glo^TM^ 9 assay kits (Promega, USA). The assay was conducted following the manufacturer’s protocols. Briefly, HepG2 cells were cultured in a 96-well plate and incubated for 24 h in a humidified atmosphere of 5% CO_2_ at 37 °C. The cells were then treated with sulforaphene (in serum free media) at an IC_50_ concentration of 72 h (33.8 μM). Cells were then incubated for 24, 48 and 72 h. After treatment, 100 μL of assay reagent was added to each well and incubated for 1 h at room temperature. Finally, the luminescence was measured using a microplate reader (Tecan Infinite M 200 PRO, Männedorf, Switzerland).

### 2.9. Cell Cycle Analysis by Flow Cytometry

The cell cycle analysis was carried out using the CycleTEST^TM^ PLUS DNA Reagent Kit (Becton Dickinson, USA) according to the protocols provided. HepG2 cells were seeded into 25 mL culture flasks at approximately 1 × 10^6^ cells/ mL and incubated for 24 h in a humidified atmosphere of 5% CO_2_ at 37 °C. Cells were treated with sulforaphene (in serum free media) at IC_50_ concentration of 72 h (33.8 μM) for 24, 48 and 72 h. Treatment-free cells were grown as a negative control. The cells were harvested with trypsin and centrifuged at 300× *g* for 10 min after treatment. Subsequently 1 mL of buffer solution (provided in the kit) was added and resuspended by gentle vortexing. The cells were centrifuged at 300× *g* for 5 min and the supernatant was discarded. A further 1 mL buffer solution was added to resuspend the cells, and following centrifugation, the supernatant was discarded. This step was repeated for a third time and cells were immediately stained with propidium iodide (200 μL). After incubation for 10 min on ice, each sample was filtered through a 12 × 75 mm polypropylene tube and analysed on the flow cytometer (Becton Dickinson, Franklin Lakes, NJ, USA).

### 2.10. Statistical Analysis

Data are presented as means ± standard deviation. Statistical evaluation was performed by two-way ANOVA using Dunnett’s test. A 95% level of confidence was considered; thus, *p* < 0.05 referred to statistical significance.

## 3. Results

### 3.1. Cytotoxicity of GRE, Sulforaphene and Cisplatin

Toxicity of intact GRE and its hydrolysed product, sulforaphene, on HepG2 cells, was determined using the MTT assay method with cisplatin as the positive control. There was no apparent cytotoxic effect of GRE for at least up to 72 h of incubation and up to a concentration of 100 μM. Sulforaphene, however, suppressed cell growth (Table 1).

Initial MTT screening with sulforaphene for 24 h indicated significant growth inhibition in HepG2 cells compared to MCF-7 and HT-29 cells (Table 2).

### 3.2. Morphological Assessment of Apoptotic Cells by DAPI Staining, AO/PI Double Staining and TUNEL Assay

**Figure 2:**
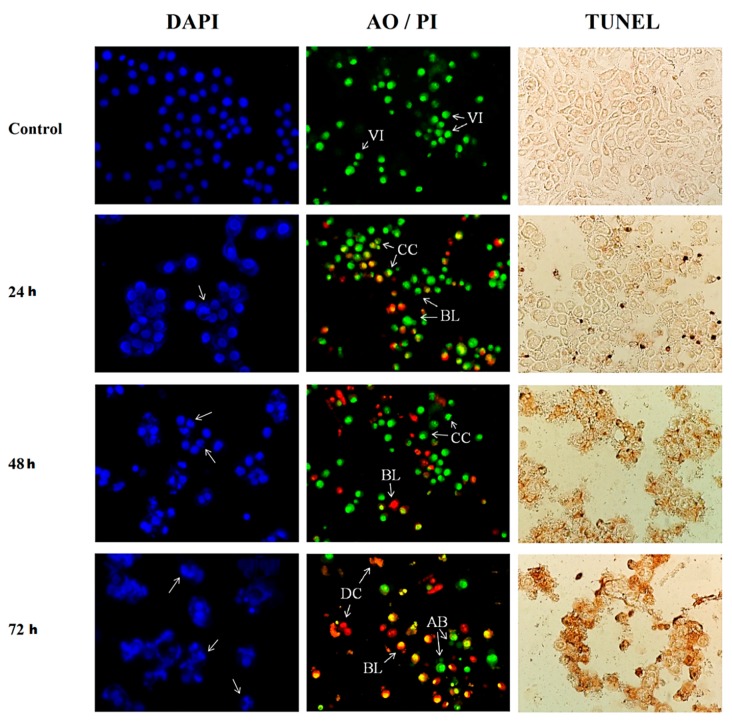
Images of stained HepG2 cells treated with sulforaphene (33.8 μM) in a time dependent manner. Cells were cultured in serum free RPMI-1640 medium and maintained at 37 °C and 5% CO_2_. DAPI fluorescence images of apoptotic HepG2 cells with arrows indicating chromatin condensation in the cell nucleus; AO/PI fluorescence images of HepG2 cells with arrows indicating viable cells (VI), chromatin condensation (CC), membrane blebbing (BL), apoptotic bodies (AB) and dead cells (DC); TUNEL assay images of HepG2 cells with darkened stains indicating DNA fragmentation within the cells. Magnification × 400; h–hours.

In general, DAPI, TUNEL and AO/PI staining methods clearly showed induction of apoptosis in HepG2 cells after treatment with sulforaphene. HepG2 cells treated with sulforaphene showed clear apoptotic features which became more apparent as the treatment period increased (Figure 2). In the DAPI staining images, there was an increase in the number of cells with small, condensed nuclei from the 24 to the 72 h treated cells, indicating an increasing number of apoptotic cells with increasing length of incubation.

Aside from nuclear condensation, DAPI staining revealed that all treated HepG2 cells visibly appeared to lose cell structure with increasing time of incubation. The control cells, however, remained intact and evenly shaped. When treated for 24 h, most cells remained intact but lost their shape once the incubation period reached 48 h. Following treatment with sulforaphene for 72 h, all cells were completely ruptured. The DAPI staining noticeably showed apoptotic morphological changes in sulforaphene treated cells in terms of both nuclear condensation and cell structure loss.

The AO/PI staining images displayed apoptotic changes in the sulforaphene treated cells in terms of colour and morphology. The apparent fluorescent colouration in the untreated cells compared to the treated cells was the first noticeable change. The control cells displayed a bright green colouration and an intact nuclear structure, indicating healthy viable cells, whereas treated cells exhibited a bright orange stain signifying the presence of apoptotic cells. With increasing incubation time with sulforaphene, the relative number of green cells gradually decreased while that of orange cells increased, indicating a shift from viable to apoptotic cells. At 72 h, the orange stain further turned to bright red indicating cell death and rupture.

Similar to the DAPI staining, AO/PI staining also revealed a change in cell structure. After 24 h of incubation with sulforaphene, there were clear indications of chromatin condensation and blebbing of the HepG2 cell membrane. After a 48-h incubation, more chromatin condensation and blebbing were observed. Upon 72 h of incubation, small apoptotic bodies and dead cells were identified. The morphological changes as well as the shift from green to orange fluorescent cells that were observed from the AO/PI staining were sequential from 24 h up to 72 h of sulforaphene treatment, indicating gradual cell death via apoptosis with increasing length of exposure.

Finally, the TUNEL assay also revealed clear apoptotic characteristics in sulforaphene-treated HepG2 cells. There was no visible colouration in untreated cells whereas in cells treated with sulforaphene, there were dark brown stains, which, similar to the other staining methods, increased in intensity with increasing duration of incubation. This change in staining denotes apparent DNA fragmentation and apoptotic nuclei while clear morphological changes in cell structure were also observed with increasing treatment period. In the period from 24 to 72 h, treated cells were observed to have gradually lost in cell integrity and eventually ruptured by the end of the incubation.

### 3.3. Annexin V-FITC Assay of Sulforaphene Treated HepG2 Cells

**Figure 3:**
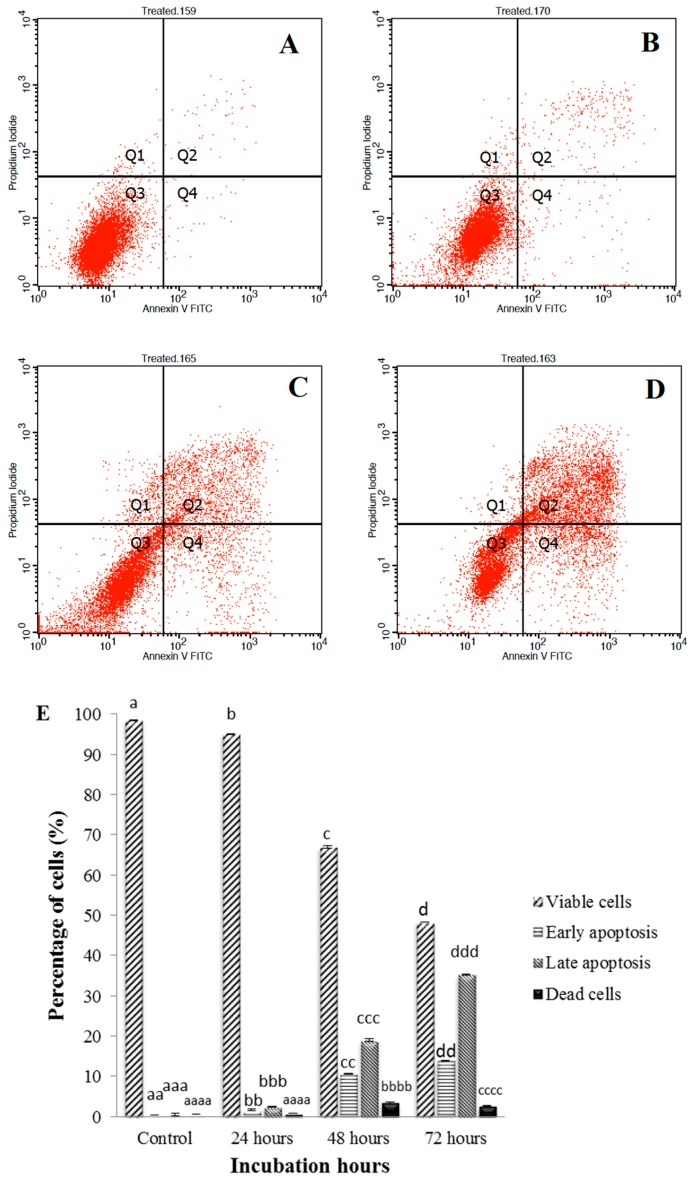
Annexin V-FITC analysis of HepG2 cells untreated (**A**) and treated with sulforaphene (33.8 μM) for 24 (**B**), 48 (**C**) and 72 (**D**) hours. Cells were cultured in serum free RPMI-1640 medium and maintained at 37 °C and 5% CO_2_. Results represent one of three independent experiments. Early apoptosis (Annexin +/PI−) is shown in the lower right quadrant (Q4) for each panel while late apoptosis (Annexin+/PI+) is shown in the upper right quadrant (Q2). Viable cells are represented in the lower left quadrant (Q3). Necrosis (Annexin −/PI+) is shown in the upper left quadrant (Q4). Chart comparison of cell distribution after sulforaphene treatment at 24, 48 and 72 h (**E**). Values are presented as means ± SD of triplicate experiments, and means with different letters differ significantly (*p* < 0.05).

The Annexin V assay revealed that sulforaphene induced early and late stage of apoptosis in HepG2 cells (Figure 3). The Annexin V-FITC plots in Figure 3 show the HepG2 cell distribution within four different quadrants (Q1, Q2, Q3, Q4) and represent one of three sets of independent experiments conducted. A uniform induction of apoptosis by sulforaphene in HepG2 cells in a time-dependent manner was observed. A decrease in viable cells was seen in all treated cells in comparison to the untreated cells. During the first 24 h of treatment, the percentage of viable cells decreased from 98.45% to 95.02%. As the treatment duration reached 48 h, the percentage of viable cells was reduced further to 66.96% and decreased even more, to 48.28%, after 72 h.

In the untreated cells, there was minimal cell distribution in Q1, Q2 and Q4 indicating a very low number of necrotic, early and late apoptotic cells, respectively. However, the cell distribution in these quadrants increased after treatment with sulforaphene. The percentage of early apoptotic cells (Q4) increased gradually with increasing treatment duration, being 1.67%, 10.58% and 13.81% following 24, 48 and 72 h of incubation, respectively. In the untreated cells, only 0.39% of cells were present in this quadrant. The untreated HepG2 cells showed a distribution of 0.60% for the late apoptotic cells (Q2). Similar to early apoptosis, the percentage of late apoptotic cells increased to 2.45%, 18.95% and 35.25% cells for 24, 48 and 72 h of sulforaphene treatment, respectively. The increase in cell distribution indicated a time dependent increase in late apoptotic cells, parallel to that noted in Q4.

Lastly, necrotic cells, represented by Q1, displayed only a slight increase in cell distribution as a result of treatment with the isothiocyanate. In the untreated cells only a percentage of 0.57% necrotic cells was observed while in the sulforaphene-treated cells it increased to 0.77%, 3.51% and 2.66% for 24, 48 and 72 h incubation, respectively. Thus, the overall cell population shift indicated that sulforaphene caused a significant increase apoptosis in HepG2 cells.

### 3.4. caspase-3/7, -8 and -9 Activity on Sulforaphene Treated HepG2 Cells

**Figure 4:**
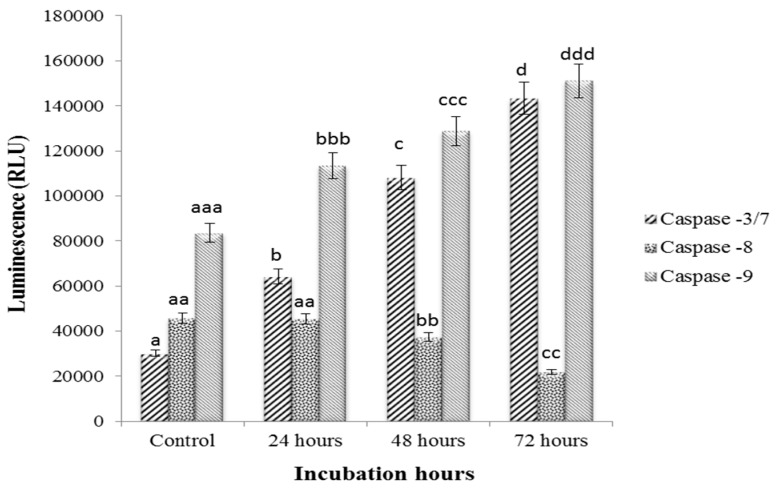
Relative luminescence expression of caspase-3/7, -8 and -9 in HepG2 cells treated with sulforaphene (33.8 μM) for 24, 48 and 72 h. Cells were cultured in serum free RPMI-1640 medium and maintained at 37 °C and 5% CO_2_. Values are presented as means ± SD of triplicate experiments, and means with different letters differ significantly (*p* < 0.05).

The treatment of HepG2 cells with sulforaphene for 24, 48 and 72 h significantly enhanced the activity of both caspase-3/7 and -9, but not caspase 8 (Figure 4).

caspase-3/7, -8 and -9 enzyme activities were detected in HepG2. caspase-3/7 and -9 enzymes were elevated upon sulforaphene treatment, the extent of increase rising with the duration of the incubation. In contrast, caspase-8 enzymes were suppressed following the same treatment, with the effect being more pronounced as the incubation period increased.

### 3.5. Cell Cycle Arrest of Sulforaphene Treated HepG2 Cells

**Figure 5:**
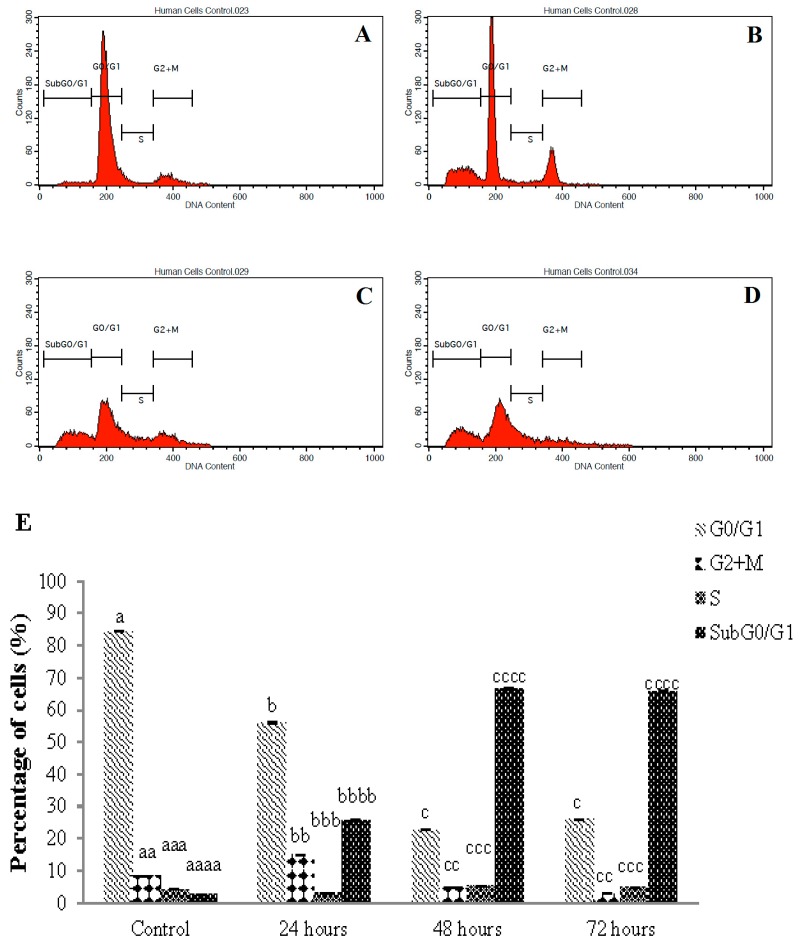
Cell cycle arrest histograms of HepG2 cells treated with sulforaphene (33.8 μM) in a time-dependent manner, and analysed by flow cytometry. Cells were cultured in serum-free RPMI-1640 medium and maintained at 37 °C and 5% CO_2_. (**A**) Untreated cells after 72 h with highest cell arrest at G_0_/G_1_ phase; Cells treated at (**B**) 24 h; (**C**) 48 h; and (**D**) 72 h. Images represent one of three independent experiments. (**E**) Chart comparison of cell cycle arrest phases after sulforaphene treatment in a time-dependent manner. Values are presented as means ± SD of triplicate experiments, and means with different letters differ significantly (*p* < 0.05).

The DNA content in sulforaphene-treated HepG2 cells was assessed using the cell cycle phase distribution analysis in a time dependent manner. The cell cycle arrest was analysed using flow cytometry, and Figure 5 shows the results of one of three independent experiments. The phases in which the cell cycle was arrested were investigated following the time-dependent exposure of HepG2 cells to sulforaphene. In the untreated cells, 84.41% of the cell population was arrested in the G_0_/G_1_ phase but decreased to 56.05% after treatment with sulforaphene for 24 h; longer treatment further reduced the number of cells arrested in this phase; however, the HepG2 cell population was seen to be increasingly arrested at the SubG_0_/G_1_ phase with increasing sulforaphene treatment period. In the control cells, a cell population of only 2.65% was arrested in the SubG_0_/G_1_ phase. This population increased to 25.74%, 66.76% and 65.86% as the sulforaphene treatment duration was extended. The arrested cell population in the G_2_+M phase decreased from 8.42% in the untreated cells, to 2.83% for the cells treated up to 72 h, while there was no significant change in cell arrest in the S phase (from 4.31% in the control sample to 4.79% in the sample treated up to 72 h).

## 4. Discussion

Over the years, our understanding of apoptosis as well as the apoptotic controlling genes in relation to cancer therapy has increased dramatically [21,22]. In addition, appreciation of the underlying molecular mechanisms has implied a more promising outcome in the treatment of cancer [23].

In particular, isothiocyanates, major derivatives of GLs, have been recognised as potential chemopreventive agents, acting through a number of mechanisms, including modulation of the various phases of cell growth [24]. Sulforaphane, the isothiocyanate derived from glucoraphanin, is one of the most recognised compounds with high anti-carcinogenic properties [25,26,27,28,29]. The current study investigated the apoptotic potential of sulforaphene, an isothiocyanate which differs from sulforaphane only by a single double bond.

The initial cytotoxicity screening showed that sulforaphene was most effective against HepG2 cells in comparison to HT29 and MCF-7 cells. Although the margin of IC_50_ values is relatively small among the three cell lines, there are other justifications for selecting HepG2 cells for further investigation. Firstly, in agreement with the present study, Sangthong et al. [30] reported the very high sensitivity of HepG2 cells towards sulforaphene, in comparison to several other compounds such as melphalan and cisplatin. Secondly, similar sensitivity towards HepG2 cells was reported for the structurally similar sulforaphane (Table 3).

Thus, HepG2 cells may be sensitive to the structurally similar sulforaphene and sulforaphane. Previous studies focused on the potential of several other isothiocyanates, such as sulforaphane, benzyl isothiocyanate, phenylethyl isothiocyanate, and erucin, to inhibit proliferation and induce apoptosis in liver cancer cells [24,31,33]. Furthermore, in Sangthong’s study [30], a comparative cytotoxic analysis of the crude extract of *Raphanus sativus* and pure sulforaphene on liver cancer cells was performed, but their effect on apoptosis was not evaluated. Hence, based on these considerations, HepG2 was selected for further investigation.

Sulforaphene exhibited an IC_50_ value of 33.8 μM when incubated with HepG2 cells for 72 h which is within the range of the value reported for most other isothiocyanates [24,33,34]. Table 3 shows published IC_50_ values for several isothiocyanates following incubation with HepG2 cells differing in terms of concentration due to a dissimilar number of cells used and the incubation hours. This indicates that sulforaphene exhibits similar cytotoxicity in HepG2 cells to the extensively studied sulforaphane and many other isothiocyanates. The parent compound, GRE, did not display cytotoxicity in these cells even following a 72 h incubation in concordance with previous studies [9,11,37,38,39,40,41,42,43].

An important attribute of chemopreventive agents is the ability to selectively induce the cell death of malignant cells via apoptosis and not through necrosis [44]. Identification of the physical self-destruction phases involved in apoptosis is used to confirm potential anticancer drugs [20,45,46]. Consistently, all morphological assessment assays in the current study revealed clear apoptotic changes in HepG2 cells treated with sulforaphene, including loss of cell integrity, condensation of cell nuclei, cell shrinkage, membrane blebbing, and the formation of apoptotic bodies, commensurate with many other studies employing various other isothiocyanates [20,47].

In validating further the apoptotic potential of sulforaphene, the annexin V-FITC assay was utilised which indicated a shift in cell distribution from viable to apoptotic after 72 h of treatment with sulforaphene, with a percentage shift of 13.81% and 35.25% cells for the early and late apoptotic stages, respectively, accompanied by a decline in cell proliferation. This cytotoxic and apoptotic potential of sulforaphene in HepG2 cells adds further to the findings by Sangthong et al. [30].

The nature of the apoptotic mechanism leading to cell death is critical in determining a promising anticancer compound. Precise modulation of caspases is crucial to avoid unusual or premature apoptotic cell death which can potentially lead to carcinogenesis, autoimmunity, neurodegeneration, and immunodeficiency [48,49]. In the current study, the parallel up-regulation of caspase-3/7 together with caspase-9 verified the involvement of the intrinsic apoptotic pathway. This type of cell death pathway commences with caspase-9 activation followed by apoptosome formation. Subsequently, after caspase-9 activation, caspase-3 and -7, the effector caspases, are then cleaved and activated [50,51]. This indicates that the treatment with sulforaphene caused mitochondrial-centred cell death, i.e., mediated by the mitochondrial outer membrane permeabilization (MOMP).

Another important attribute of a potential anticancer agent is the ability to initiate cell cycle arrest in cancer cells [52]. The molecular mechanism underlying the cytotoxicity of a compound may be understood from the analysis of the cell cycle arrest phase [19]. For instance, cell death, resulting from DNA fragmentation, produces apoptotic cells with less DNA than healthy cells, which brings about a subG_0_/G_1_ peak in a cell population profile [53]. Corresponding to this finding, in the present study, sulforaphene induced subG_0_/G_1_ phase arrest in HepG2 cells, suggesting a reduced DNA content. The detection of subG_0_/G_1_ phase arrest occurs only if sufficient cellular DNA has been lost. Thus, sulforaphene has the potential to cause sufficient DNA loss to halt the cell cycle at the subG_0_/G_1_ phase in HepG2 cells.

The subG_0_/G_1_ phase arrest in HepG2 cells alludes to a possible relationship with the intrinsic death pathway as indicated by the up-regulation of caspase-3/7 and -9 that may be linked to the generation of reactive oxygen species as similarly indicated by Papi et al. [19]. According to Angelino and Jeffery [54] the bioavailability of GLs hydrolysis products contributed to their efficacy in cancer prevention. In our study, the conversion of GRE to its bioactive compounds might be responsible for the biological activity; thus, the bioavailability of GRE merits further investigation.

## 5. Conclusions

This article focuses on a study of the cytotoxicity and apoptotic potential of sulforaphene in HepG2 cells. Sulforaphene was cytotoxic in these cells whereas its precursor, the glucosinolate GRE, was inactive. Morphological analysis employing AOPI, DAPI and TUNEL staining assays revealed clear apoptotic activity. In further studies using Annexin V-FITC and caspase, analyses established significant increases in early and late apoptosis, and in the activity of caspases -3/7 and -9. Moreover, impairment of cell proliferation by cell cycle arrest at the Sub G_0_/G_1_ phase as compared to the G_0_/G_1_, G_2_ + M and S phases was noted.

The present findings suggest positive apoptotic activity of sulforaphene in HepG2 cells via successive accompaniment of apoptosis subsequent to cell proliferation inhibition by the compound. These findings will contribute to the effort to identify phytochemicals that can potentially function as human anticancer agents.

## Figures and Tables

**Table 1:** Cytotoxicity of GRE, sulforaphene and cisplatin in HepG2 cells.

Compound	IC_50_ (μM)		
	24 h	48 h	72 h
Glucoraphenin (GRE)	ND	ND	ND
Sulforaphene	40.1 ± 0.41	38.5 ± 0.14	33.8 ± 0.52
Cisplatin	7.5 ± 0.12	2.0 ± 0.04	0.7 ± 0.00

ND: not detected.

**Table 2:** Cytotoxicity of sulforaphene in HepG2, MCF-7 and HT-29 cells.

Compound	IC_50_ (μM)		
	HepG2	MFC-7	HT-29
Sulforaphene	40.0 ± 0.08 ^a^	41.1 ± 0.08 ^a,b^	42.3 ± 0.05 ^b^

Values are presented as means ± SD of triplicate experiments, and means with different letters differ significantly (*p* < 0.05).

**Table 3:** Cytotoxicity of several isothiocyanates against HepG2 cell line from various studies.

ITCs	Incubation Hours	IC_50_ Value (μM)	References
Glucoraphanin ITC	48	6.13	[31]
Benzyl ITC	72	7.30	[24]
Allyl ITC	24	10.00	[32]
Phenethyl ITC	72	11.20	[24]
Glucoraphanin ITC	72	12.80	[24]
Glucoerucin ITC	24	23.18	[33]
β-phenylethyl ITC	24	24.60	[34]
Sulforaphene	72	33.80	Current research
Allyl ITC	72	35.50	[24]
Glucoraphanin ITC	24	65.20	[35]
Glucoraphenin ITC	72	79.65 *	[30]
Glucomoringin ITC	NA	192.71 *	[36]

* Converted value from μg/mL; NA: not available.
